# Intersectional Analysis of Suicide-related Emergency Department Visits in Youth in California, 2018–2021

**DOI:** 10.5811/westjem.47097

**Published:** 2025-11-26

**Authors:** Laura M. Prichett, Annie Na, Hanae Fujii-Rios, Emily E. Haroz

**Affiliations:** * Johns Hopkins University School of Medicine, Department of Pediatrics, Division of General Pediatrics, Baltimore, Maryland; † Johns Hopkins Bloomberg School of Public Health, Center for Suicide Prevention, Baltimore, Maryland; ‡ Johns Hopkins Bloomberg School of Public Health, Department of International Health, Baltimore, Maryland; § Johns Hopkins University School of Medicine, Department of Pediatrics, Division of Pediatric Emergency Medicine, Baltimore, Maryland; || Johns Hopkins Bloomberg School of Public Health, Department of International Health, Social and Behavioral Health Program, Center for Indigenous Health, Baltimore, Maryland

## Abstract

**Introduction:**

The COVID-19 pandemic and related anti-Asian political rhetoric had a detrimental impact on the mental health of Asian American and Pacific Islander (AAPI) youth in the United States. Our objective was to quantify trends in suicide-related emergency department (ED) encounters among AAPI youth before and during the COVID-19 pandemic, using an intersectional lens of race and sex and to contextualize these trends on a timeline of political and social events (such as anti-Asian hate crimes) occurring during the same period in California.

**Methods:**

Using data from the California State Emergency Department Database (SEDD) from 2018–2021, we evaluated changes in quarterly proportions of suicide-related ED encounters by age, race, and sex subgroups by comparing mean percentage change in proportions before and during the pandemic among patients 8–21 years of age. We evaluated changes in quarterly proportions of suicide-related ED encounters by age, race, and sex subgroups by comparing mean percentage changes as they related to events around the pandemic and spikes in anti-Asian hate crimes. To compare relative disparities during the periods, we used stratified adjusted mixed multilevel logistic regression, with White males as the reference group.

**Results:**

The overall increase in suicide-related ED visits for all youth during this period was 49.5% (95% CI 46.7–52.2%), representing 2,637 more suicide-related ED visits in 2021 than 2018. The graphical observational analysis of changes in quarterly proportions of suicide-related ED visits showed some temporal correlation between spikes in rates among AAPI and American Indian and Alaska Native (AI/AN) females and specific events, such as anti-Asian hate crimes and school closings. The largest percentage increase was seen among females of all races, and in particular, AI/AN females (+58.1%, representing 471 more suicide-related ED visits in 2021 than 2018) and AAPI females (+57.5%, representing 1,545 more suicide-related ED visits in 2021 than 2018). During the pandemic, the adjusted odds of a suicide-related ED visit among AAPI females 13–17 years of age compared to White males was 2.01 (95% CI, 1.91–2.13). A total of 131 in-ED deaths occurred during the study period, with no significant year-to-year variation in the number of deaths.

**Conclusion:**

Suicide-related ED visits increased for all youth during COVID-19, with the sharpest rise among AAPI and AI/AN females. Asian American and Pacific Islander females 8–12 and 13–17 years of age showed especially large increases. While causality cannot be inferred, patterns aligned with pandemic disruptions and anti-Asian hate crimes. Findings highlight the value of intersectional analysis to identify disproportionately impacted subgroups and inform future, culturally responsive suicide prevention efforts.

## INTRODUCTION

The COVID-19 pandemic and related anti-Asian political rhetoric had a markedly detrimental impact on the mental health of people who identify as Asian American and Pacific Islander (AAPI) in the United States.^1^–^4^ The rate of hate crimes against AAPI in the US increased considerably during the pandemic as compared to pre-pandemic years^5^; one national survey found that 60.7% of Asian Americans experienced at least one incident of verbal or physical discrimination between 2020–2021, and that being Asian American, female, and younger were some of the factors associated with increased discrimination.^6^–^7^ According to a national poll conducted in April 2020, approximately 30% of all respondents witnessed an Asian person being “verbally blamed” for the pandemic.^8^

In parallel, emergency department (ED) visit rates for self-harm in adolescents and young adults exceeded that of middle-aged adults by approximately three-fold in 2020, and ED encounters for suicidal ideation and attempts among young people increased five-fold between 2011–2021.^9^–^10^ These and other troubling indicators led the American Academy of Pediatrics to declare a national emergency in child and adolescent mental health in 2021, noting the disproportionate impact of the pandemic on ethnically and racially minoritized youth.^11^ Adolescent females, in particular, have seen increases in rates of depression, anxiety, and suicidal thoughts and behaviors both before and during the pandemic.^10^,^12^–^15^ For this reason, we used an intersectional lens in this analysis—an important tool in highlighting experiences and needs unique to those belonging to multiple marginalized groups, such as ethnoracially minoritized females.^14^,^16^,^17^

Among AAPI adults, racial discrimination has been associated with post-traumatic stress disorder, suicidal ideation, and all-cause mortality.^2^,^3^,^18^ In a survey study of 1,697 AAPI university students in the US, discrimination related to COVID-19 was associated with higher odds of self-injury and suicidal ideation.^19^ Despite this, little attention has been paid specifically to how the pandemic may have impacted the mental health of AAPI children and adolescents. To address these troubling trends, it is critical to understand the full impact of the COVID-19 pandemic on the mental health of all young people, especially subgroups such as young male and female AAPI in the US, who have previously not been considered high risk for suicidal thoughts and behaviors and yet have been deeply impacted by targeted, politically charged racism.^7^,^20^,^21^

In this work, we sought to quantify trends in suicide-related emergency department (ED) encounters among male and female young people from various racial and ethnic groups during the period of the COVID-19 pandemic. Our study is among the first to use a large, statewide ED dataset to conduct an intersectional analysis of suicide-related ED visits by race and sex, with a specific focus on AAPI youth. Most prior studies report aggregated racial groups or do not disaggregate trends by age-sex-race subgroups. Our stratified, longitudinal approach can identify disproportionately affected populations that may be obscured in more aggregated analyses. To this end, we used ED data from California, the state with the largest AAPI population in the US.^22^ We used data from before COVID-19 (2018 and 2019) and during COVID-19 (2020 and 2021) to understand trends in suicide-related ED visits as they relate to historic events during this time, specifically quarantines, mandated statewide school closures, and marked increases in hate crimes against AAPI individuals in California that occurred in the spring and summer of 2020.^23^

**Population Health Research Capsule**

What do we already know about this issue?

*Youth mental health worsened during COVID-19, with rising emergency department visits for suicidal thoughts and behaviors and widening disparities across minoritized populations*.

What was the research question?

*How did suicide-related ED visits among Asian and other minoritized youth change during COVID-19 across race and sex groups?*

What was the major finding of the study?

*Asian American and Pacific Islander (AAPI) girls had 2.01 times higher odds of suicide-related ED visits during COVID-19 than White boys (95% CI, 1.91–2.13, P < 001)*.

How does this improve population health?

*Spotlighting disparities identified youth subgroups at highest suicide risk during COVID-19 to inform targeted, culturally responsive prevention strategies*.

## METHODS

### Data Source and Sample

The ED visit data was derived from the Agency for Healthcare Research and Quality (AHRQ) Healthcare Cost and Utilization Project (HCUP) California State Emergency Department Database (SEDD) from 2018–2021. The SEDD is a complete registry of ED encounters that do not result in an admission and includes diagnoses and procedures as well as patient demographics (eg, age, race, ethnicity, sex), insurance information, and total amounts billed. For this study, we identified ED visits for patients 8–21 years of age. Detailed information on the SEDD is available on the HCUP website.^24^ This study was determined to be exempt from full institutional review board (IRB) review, by the institution’s School of Medicine IRB.

### Case Definitions

We classified age groups as 8–12, 13–17, and 18–21 years of age. The lower bound of eight years was chosen due to small numbers of suicide-related ED events among younger children in the data, making stratified analyses infeasible. We classified race and ethnicity according to the groupings within the SEDD, which included: “White,” “Black,” “Hispanic,” “Asian or Pacific Islander” (identified here using the acronym AAPI), “Native American,” and “other.” For purposes of the analysis, we did not examine the grouping of “other,” as we did not have details as to who may have been included in this group. The SEDD classifies sex only as “male” or “female”; accordingly, these groupings were used in this analysis, and the term “sex” is used throughout, noting that we do not actually have information about biological sex or gender identity.

We generated intersectional subgroups by combining sex and racial/ethnic group (ie, AAPI female, AAPI male, White female, White male, etc). Two proxies for socioeconomic status were used: 1) primary insurance, classified as “private,” “public,” or “none/other”; and 2) median income per patient’s ZIP Code. We used *International Classification of Diseases, 10^th^. Modification* (*ICD-10*) diagnosis codes to identify suicide-related encounters. We chose to use a broad classification to encompass all potential suicide-related billing diagnoses including suicidal ideation (*ICD-10* code: R45851), suicide attempt ,and/or intentional self-harm with suicidal intent (*ICD-10* codes: T36–T65, X71–X83, T1491, T71112A-T71232S, X710XXA-X838XXS, Z915).^9^,^24^

### Data Analysis

#### Graphical Analysis

We conducted an observational, descriptive analysis of temporally aligned trends in suicide-related ED encounters and key political and social events during the years of the COVID-19 pandemic. The percentage of all ED encounters with diagnosis codes for suicidal ideation, suicidal behavior, and/or self-harm with suicidal intent were calculated by race/sex subgroup as well as age subgroup. We used interclass correlation coefficients (ICC) to determine whether denominators (all ED visits) differed by subgroup from quarter to quarter and year to year, to ensure that trends in the proportion of suicide-related encounters were not biased due to changing denominators of ED visits from year to year. We compared characteristics of patients with and without suicide-related ED visits using chi-squared tests for categorical variables and Welch two-sample *t*-tests for continuous variables. Graphical representations of trends in quarterly proportions of suicide-related encounters were generated by race and age group, including markers for dates of specific policy changes in California, including quarantine, reported increases in racially motivated hate crimes, school closures, and vaccine availability for adolescents.

#### Analysis of Percentage Change

We compared the mean quarterly change by race and sex subgroups before and during the pandemic using Welch two-sample *t*-tests. The period before the COVID-19 pandemic was classified as Q1 of 2018 through and including Q1 of 2020, and the period during the COVID-19 pandemic was classified as Q2 of 2020 through and including Q4 of 2021.^25^ We performed stratified analysis by sex and racial/ethnic subgroup as well as age groups.

#### Relative Disparities Regression Analysis

We used a series of unadjusted and adjusted mixed multilevel logistic regression models, stratified by pre- and during-COVID-19 periods, to understand differences in the relationship between suicide-related ED visits between sex and race subgroups. We used the White male subgroup as the reference category for three reasons: 1) this subgroup’s historical prominence in suicide research and prevention frameworks; 2) consistently elevated suicide death rates relative to other groups; and 3) lower exposure to racism-related stressors during the pandemic.^26^ This framing allows clearer contrast with subgroups that may have been experiencing disproportionate increases. As a result, the odds ratios indicated the odds of a suicide-related ED encounter for AAPI females, AAPI males, White females, etc, as compared to White males separately before and during the pandemic. Adjusted models included insurance type and household income quartile; hospital was treated as a nested level to account for potential clustering by hospital. Non-overlapping confidence intervals for the pre-during time periods indicated a significant change in odds between the periods, relative to White males. All statistical tests were two sided, with a *P* value ≤ .05 considered significant. We performed all statistical analyses using R software, v4.3.2 (The R foundation for Statistical Computing, Vienna, Austria) and RStudio software, v2023.12.0 (Posit PBC, Boston, MA).

## RESULTS

### Overall Trends in the Total Study Sample, 2019–2022

The proportion of ED visits that were suicide-related in this population ranged from 3.3% in 2019 to 4.7% in 2021 (Table 1). The median age across all years was 16 (interquartile range 14,19), and the age category 13–17 years of age had the highest number of suicide-related ED visits as compared to all other age groups (50.0%, n = 116,754; Table 1). There were more suicide-related ED visits by females than males (60.9% vs 39.1%), and suicide-related ED visits were the most common within the Hispanic subgroup and White subgroup during the study period, although it should be noted that these groups had the highest number of ED visits overall (Table 1). When stratified by race and sex, the highest proportion of suicide-related ED visits overall were among White females (6.3%), Asian females (5.3%), and White males (4.8%) (Table 1). The highest proportion of suicide-related ED visits was among patients with either private insurance (5.2%) or Medicare (4.2%), or as the expected primary payor (Table 1). The ICC analysis indicated that the overall number of ED visits by race/ethnic and sex subgroup did not change significantly from year to year and, thus, was not the underlying reason for trends in the proportion of suicide-related ED encounters.

### Graphical Analysis

**Figure 1:**
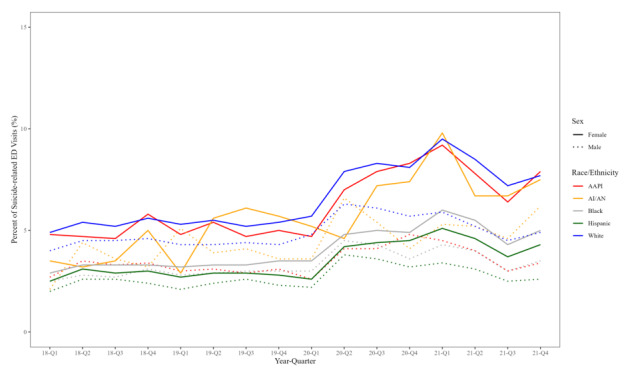
Quarterly trends in suicide-related emergency department visits by race and sex, 8–21 years of age, 2018–2021. *ED*, emergency department; *AAPI*, Asian American Pacific Islander; *AI/AN*, American Indian and Alaska Native.

**Figure 2:**
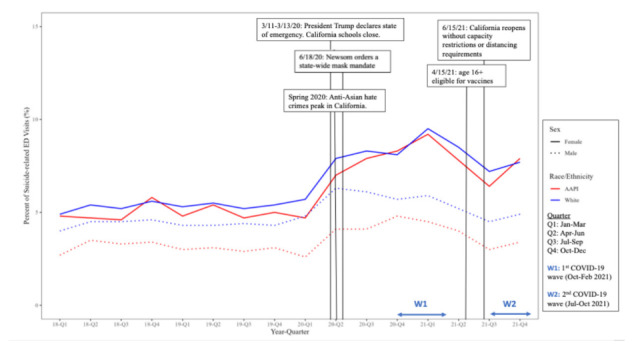
COVID-19-related annotated quarterly trends in suicide-related emergency department visits in California among AAPI and White males and females, 8–21 years of age, 2018–2021. *ED*, emergency department; *AAPI*, Asian American Pacific Islander.

In the graphical analysis of quarterly trends in the proportion of suicide-related ED visits during the study period, there was a notable increase in all subgroup rates in the second quarter of 2020, coinciding with the beginning of the COVID-19 pandemic. Before the second quarter of 2020, AAPI, American Indian and Alaska Native (AI/AN), and White females had the highest proportions of suicide-related ED visits, and by the end of 2020, ED visits among these groups peaked at 10%, increasing the gap between the three groups and the other subgroups (Figure 1). While all subgroup rates increased during the COVID-19 pandemic period, the highest proportions of suicide-related ED visits were among AAPI and White females. When compared to a contextual overlay of social and political events at that time, the rates track with statewide school closures and mask mandates, as well as anti-Asian hate crimes in California, which increased by 162% in 2020 as compared to the average number of hate crimes over the previous four years.^23^ The proportions leveled off closer to 7% in late 2021, which corresponds with the time when vaccines became available for adolescents and schools reopened (Figure 2).

### Analysis of Percentage Change

The overall increase in suicide-related ED visits for all youth during this period was 49.5% (95% CI, 46.7–52.2%), representing 2,637 more suicide-related ED visits in 2021 than 2018. Among all ages, the largest increases in mean quarterly percentage of suicide-related ED visits were seen among females and ranged from an increase of 52.6% among White females to a 58.1% increase among AI/AN females, with AAPI females demonstrating the second highest increase of 57.5% (Table 2). Comparatively, the increases in mean quarterly percentage of suicide-related ED visits among males ranged from an increase of 25.5% among White males to a 43.1% increase among AI/AN males, with AAPI males having the second lowest increase of 28.8%. There is notable variation by age, among youth 8–12 years of age. Black females saw an increase of 103.2%, representing a more than double increase between the two time periods, while AAPI females saw an increase of 91.6%, which was the second-largest increase in this age group. Among youth 13–17 years of age, the greatest increase was seen among AI/AN males (90.6%), but the second through sixth largest increases were all among females, with the second largest increase among AAPI females (77.6%). Among youth 18–21 years of age, the increases were not as dramatic, with most in the 20% range, although AI/AN females saw an increase of 62.1% (Table 2).

### Relative Disparities Regression Analysis

In the adjusted analysis, most male racial and ethnic subgroups of youth were as likely or less likely than White males to have a suicide-related ED encounter, both before and during the pandemic (Table 3). White, AAPI and AI/AN females generally had increased odds of an ED encounter both before and during the pandemic and, with the exception of AI/AN females, the confidence intervals for the pre- and during periods did not overlap, indicating a significantly increased difference in odds of a suicide-related ED encounter during the pandemic. The most pronounced odds of suicide-related ED encounters occurred among youth 13–17 years of age during the pandemic, as AAPI and White females were more than twice as likely to have an ED encounter for a suicide-related cause as White males (AAPI females, OR 2.01, 95% CI, 1.91–2.13; White females, OR 2.21, 95% CI, 2.15–2.29).

## DISCUSSION

In 2020, a study by Lo et al used the AHRQ Nationwide Emergency Department Sample (NEDS) and methods similar to those in this work to determine that between 2007–2016 there was a five-fold increase in the proportion of suicide-related ED visits among young people.^27^ The trends we found raise similar concern about accelerating rates of suicide-related ED visits among adolescents in California over a short time frame. The lack of available longitudinal race data in the NEDS made it impossible to explore racial differences in suicide-related ED visit trends, an important step in fully understanding the landscape of suicide prevention needs. In this study, we used the SEDD dataset to explore racial differences in suicide-related ED visits.

Although suicide-related ED visit proportions increased for all racial/ethnic subgroups during COVID-19, we found that AAPI and AI/AN females had the greatest percentage increase across all ages combined. The mean percentage of suicide-related ED visit rates for AAPI females almost doubled for those 8–12 years of age during the pandemic, and the greatest increase was seen among youth 13–17 years of age. This is consistent with previous research findings that young AAPI females suffered disproportionately from racism during the period of the COVID-19 pandemic.^7^ The proportions of suicide-related ED encounters among AAPI females tracked closely not only with COVID-19-related school closures and isolation orders but also with the uptick in anti-Asian hate crimes in California in 2020 (Figure 2).^23^

It is clear from the graphical analysis presented in Figure 1 that the mental health of all youth was negatively impacted during the COVID-19 pandemic, regardless of sex and race or ethnicity. Our analysis focused on the outcome of suicide-related ED encounters, as opposed to death by suicide to more broadly capture mental health indicators specific to females, a group generally less likely to die by suicide than males, but more likely to suffer from depression and anxiety.^29^–^31^ We additionally found that after adjusting for insurance type and median household income quartiles, the odds of having suicide-related ED encounters increased during the pandemic universally among females as compared to those before the pandemic. This is consistent with previous findings that females from all racial/ethnic groups may have experienced a greater overall negative emotional impact as a result of the pandemic than male peers,^7^,^15^,^32^–^35^ as well as increased suicide-related ED visits during the pandemic.^36^ While the acute phase of the pandemic has ended, the mental health effects on youth, particularly among marginalized groups, are ongoing. Suicide-related ED visits remain elevated, and our findings highlight youth populations (eg, AAPI females, AI/AN youth) who may have been under-recognized.

## LIMITATIONS

It is important to acknowledge the limitations associated with using clinical data, such as the SEDD, for this type of research. The SEDD only includes patients discharged from the ED, excluding those admitted to the hospital who may have more serious presentations of suicide risk. We found that while AAPI and AI/AN males had the lowest proportions of suicide-related ED visits (2.1% and 0.2%, respectively), these two groups had the highest mortality rates after presentation to the ED (14.1 and 24.3 per 100,000, respectively; Supplemental Table 1). That said, mortality data from the SEDD should be interpreted with caution, as these data would only have captured the subset of patients who were admitted to the ED alive but died subsequently.^24^ Neither do the SEDD’s data elements include suicide risk screening information (such as the ASK [ask suicide-screening questions] tool), leaving opportunities for missing individuals who may have screened positive for suicide risk but did not have an *ICD-10* code related to suicide risk.^24^ Finally, *ICD-10* codes for intentional self-harm may include some accidental self-harm events, particularly among younger children, potentially inflating suicide-related visit estimates.^37^,^38^

The SEDD only collects sex and gender data as “male” or “female,” which did not allow for the exploration of suicide-related ED encounters among transgender and non-gender conforming youth in this analysis; this is an important area for future research. Similar to many other large datasets, the SEDD’s categories of race and ethnicity do not allow for a more granular focus on the many heterogenous groups that make up each of the racial subgroups used in this analysis. The grouping AAPI, in particular, represents a wide array of cultural backgrounds, languages, immigration histories, generational statuses, and belief systems, and this certainly represents an area for further study.^6^ It should be noted that causal inferences cannot be drawn from our graphical analysis of temporal associations between social and political events during the pandemic and suicide-related ED encounters. However, the temporal alignment between public events (eg, spike in anti-Asian hate crimes) and ED visit trends offers a hypothesis-generating basis for future studies that incorporate direct measures of discrimination and longitudinal mental health outcomes.

We believe the strengths associated with using SEDD data in this work far outweigh these limitations. The SEDD is a large, longitudinal data source containing detailed information on patient demographics, diagnosis codes and disposition, and has very few missing data.^24^ The California SEDD, in particular, represents a large, diverse patient population, providing enough statistical power for our disaggregated analysis. While this paper primarily focuses on the AAPI population, it is worth stressing the need for more research into the mental health of young females in general and specific minority groups such as Black females 8–12 years of age, among whom the proportion of suicide-related ED encounters more than doubled (103%) during the COVID-19 pandemic. Similar to the AAPI population during the pandemic, Black Americans faced increased stress during the pandemic related to concurrent events, such as media attention surrounding Black Lives Matter and police violence against unarmed Black citizens.^39^–^42^

## CONCLUSION

Broadly speaking, a more complete understanding of the intersectional epidemiology of suicide risk is an important step toward preventing suicide-related ED encounters and addressing the national youth mental health crisis. The 2024 National Strategy for Suicide Prevention^43^ provides an actionable framework for policymakers to enact a wide range of upstream and downstream, evidence-based suicide-prevention solutions, from large-scale efforts like infrastructure building and prioritizing equity to more specific efforts such as increasing mental health services capacity, lethal means reduction, and crisis care accessibility. Legislators should support continued funding of the 988 platform and related communication programs, and health systems should ensure that systems are in place to identify, support, and appropriately follow up with patients at risk for suicide. Our findings support the prioritization of intensive, multifaceted, culturally sensitive approaches to suicide prevention research and mental health programming to ensure that all young people with mental health needs are identified and provided with the best possible care.

## Supplementary Information





## Figures and Tables

**Table 1:** Characteristics of the study population by suicide-related and non-suicide-related visits.

Characteristics	All ED Visits N	Suicide-related ED Visit 1 n (%)	Non-Suicide-related ED Visit 1 n (%)
N	6,050,870	233,448%	5,817,422%
Age	16.0 (12.0, 19.0)	16.0 (14.0, 19.0)	16.0 (12.0, 19.0)
Age (Categorical)			
8–12 years	1,626,796 (26.9)	31,614 (13.5)	1,595,182 (27.4)
13–17 years	1,991,371 (32.9)	116,754 (50.0)	1,874,617 (32.2)
18–21 years	2,432,703 (40.2)	85,080 (36.4)	2,347,623 (40.4)
Sex			
Female	3,270,817 (54.1)	142,076 (60.9)	3,128,741 (53.8)
Male	2,780,053 (45.9)	91,372 (39.1)	2,688,681 (46.2)
Race			
AAPI	298,190 (4.9)	13,731 (5.9)	284,459 (4.9)
AI/AN	22,213 (0.4)	1,068 (0.5)	21,145 (0.4)
Black	607,471 (10.0)	21,789 (9.3)	585,682 (10.1)
Hispanic	3,482,055 (57.5)	104,837 (44.9)	3,377,218 (58.1)
White	1,640,941 (27.1)	92,023 (39.4)	1,548,918 (26.6)
Insurance			
Medicare	84,611 (1.4)	3,530 (1.5)	81,081 (1.4)
Medicaid	3,562,367 (58.9)	114,508 (49.1)	3,447,859 (59.3)
Private Insurance	1,855,930 (30.7)	96,587 (41.4)	1,759,343 (30.2)
Self-Pay/ Other/ None/ Unknown	547,962 (9.1)	18,823 (8.0)	529,139 (9.1)
Race/Sex			
AAPI, male	149,067 (2.5)	4,964 (2.1)	144,103 (2.5)
AAPI, female	149,123 (2.5)	8,767 (3.8)	140,356 (2.4)
AI/AN, male	9,803 (0.2)	415 (0.2)	9,388 (0.2)
AI/AN, female	12,410 (0.2)	653 (0.3)	11,757 (0.2)
Black, male	262,297 (4.3)	8,350 (3.6)	253,947 (4.4)
Black, female	345,174 (5.7)	13,439 (5.8)	331,735 (5.7)
Hispanic, male	1,608,210 (26.6)	41,969 (18.0)	1,566,241 (26.9)
Hispanic, female	1,873,845 (31.0)	62,868 (26.9)	1,810,977 (31.1)
White, male	750,676 (12.4)	35,674 (15.3)	715,002 (12.3)
White, female	890,265 (14.7)	56,349 (24.1)	833,916 (14.3)
Median Household Income Quartile			
Q1	2,176,204 (36.0)	65,107 (27.9)	2,111,097 (36.3)
Q2	1,735,094 (28.7)	64,882 (27.8)	1,670,212 (28.7)
Q3	1,262,816 (20.9)	57,189 (24.5)	1,205,627 (20.7)
Q4	876,756 (14.5)	46,270 (19.8)	830,486 (14.3)
Year			
2018	1,763,631 (29.1)	59,646 (25.6)	1,703,985 (29.3)
2019	1,761,692 (29.1)	58,698 (25.1)	1,702,994 (29.3)
2020	1,192,620 (19.7)	52,821 (22.6)	1,139,799 (19.6)
2021	1,332,927 (22.0)	62,283 (26.7)	1,270,644 (21.8)

1 *P*-value not shown. All differences were significant at the *P* < .001 level.

Pearson chi-squared test was used for categorical variables, and Welch two-sample *t*-test for continuous variables.

*ED*, emergency department; *AAPI*, Asian American Pacific Islander; *AI/AN*, American Indian and Alaska Native.

**Table 2:** Mean quarterly percentage of suicide-related emergency department encounters by race/ethnicity and age group before and during the COVID-19 pandemic (2018–2021, N = 6,050,870).

	Pre-COVID-19		During COVID-19		% Change	P-value^1^
	
	Mean	95% CI	Mean	95% CI		
All Ages (8–21)						
AAPI, male	3.09	2.85, 3.33	3.98	3.40, 4.56	28.80	.008
AAPI, female	4.94	4.64, 5.24	7.78	6.95, 8.61	57.49	< .001
AI/AN, male	3.74	3.10, 4.37	5.35	4.58, 6.12	43.05	.002
AI/AN, female	4.51	3.56, 5.47	7.13	5.70, 8.56	58.09	.004
Black, male	2.87	2.73, 3.00	3.89	3.38, 4.40	35.54	.002
Black, female	3.30	3.17, 3.42	5.06	4.55, 5.58	53.33	< .001
Hispanic, male	2.37	2.20, 2.53	3.19	2.75, 3.63	34.60	.003
Hispanic, female	2.83	2.68, 2.97	4.41	4.02, 4.80	55.83	< .001
White, male	4.40	4.22, 4.57	5.52	4.90, 6.15	25.45	.004
White, female	5.36	5.16, 5.56	8.18	7.51, 8.85	52.61	< .001
Ages 8–12						
AAPI, male	1.24	1.00, 1.47	1.43	0.89, 1.98	15.32	.44
AAPI, female	2.03	1.80, 2.27	3.89	2.99, 4.78	91.63	.002
AI/AN, male	2.28	1.31, 3.25	2.54	1.27, 3.81	11.40	.71
AI/AN, female	3.10	0.90, 5.29	4.15	2.39, 5.91	33.87	.39
Black, male	1.69	1.49, 1.90	2.57	2.00, 3.14	52.07	.008
Black, female	1.86	1.67, 2.05	3.78	2.98, 4.57	103.23	< .001
Hispanic, male	1.24	1.00, 1.47	1.53	1.31, 1.76	23.39	.05
Hispanic, female	1.59	1.43, 1.76	2.85	2.45, 3.26	79.25	< .001
White, male	2.18	1.93, 2.43	2.70	2.34, 3.06	23.85	.02
White, female	2.64	2.38, 2.90	4.94	4.41, 5.47	87.12	< .001
Ages 13–17						
AAPI, male	4.08	3.66, 4.51	5.51	4.25, 6.78	35.05	.03
AAPI, female	7.55	6.93, 8.18	13.41	11.49, 15.34	77.62	< .001
AI/AN, male	4.37	3.59, 5.14	8.33	7.22, 9.45	90.62	< .001
AI/AN, female	7.50	5.95, 9.04	11.21	9.10, 13.33	49.47	.005
Black, male	3.48	3.22, 3.73	4.85	4.02, 5.68	39.37	.006
Black, female	5.65	5.33, 5.97	9.23	8.12, 10.35	63.36	< .001
Hispanic, male	3.02	2.91, 3.13	3.96	3.29, 4.62	31.13	.01
Hispanic, female	4.62	4.41, 4.84	7.75	6.87, 8.64	67.75	< .001
White, male	5.64	5.31, 5.98	7.14	6.11, 8.17	26.60	.01
White, female	8.77	8.29, 9.26	14.23	12.75, 15.71	62.26	< .001
Ages 18–21						
AAPI, male	3.99	3.75, 4.24	4.46	3.81, 5.11	11.78	.14
AAPI, female	4.68	4.39, 4.98	5.62	5.34, 5.91	20.09	< .001
AI/AN, male	4.34	3.47, 5.20	4.51	2.74, 6.27	3.92	.84
AI/AN, female	3.09	2.10, 4.09	5.01	3.64, 6.38	62.14	.02
Black, male	3.33	3.10, 3.55	3.99	3.64, 4.35	19.82	.003
Black, female	2.63	2.49, 2.77	3.51	3.20, 3.83	33.46	< .001
Hispanic, male	2.99	2.85, 3.14	3.64	3.26, 4.02	21.74	.005
Hispanic, female	2.19	2.06, 2.32	2.75	2.60, 2.90	25.57	< .001
White, male	5.01	4.73, 5.29	5.84	5.32, 6.35	16.57	.007
White, female	4.16	4.00, 4.33	5.18	4.74, 5.63	24.52	< .001

*CI*, confidence interval; *AAPI*, Asian American Pacific Islander; *AI/AN*, American Indian and Alaska Native.

**Table 3:** Odds of suicide-related emergency department encounter by race/ethnicity and age group before and during the COVID-19 pandemic (2018–2021, N = 6,050,870). Mixed multilevel logistic regression analysis, hospital as nested level

	Pre-COVID-19 (2018–2019)		During COVID-19 (2020–2021)	
	
	OR (95% CI)	aOR† (95% CI)	OR (95% CI)	aOR† (95% CI)
All Ages (8–21)				
White, male	Reference	Reference	Reference	Reference
White, female	1.24 (1.22–1.26)**	1.23 (1.21–1.25)**	1.56 (1.52–1.59)**	1.56 (1.52–1.59)**
AAPI, male	0.69 (0.66–0.72)**	0.71 (0.68–0.74)**	0.69 (0.66–0.72)**	0.70 (0.67–0.74)**
AAPI, female	1.12 (1.08–1.15)**	1.13 (1.09–1.16)**	1.43 (1.38–1.48)**	1.46 (1.41–1.52)**
AI/AN, male	0.94 (0.82–1.07)	0.98 (0.86–1.12)	1.05 (0.90–1.22)	1.15 (0.98–1.34)
AI/AN, female	1.13 (1.01–1.25)*	1.19 (1.07–1.32)*	1.43 (1.26–1.62)**	1.49 (1.31–1.69)**
Black, male	0.68 (0.66–0.70)**	0.77 (0.75–0.80)**	0.70 (0.67–0.73)**	0.80 (0.77–0.84)**
Black, female	0.79 (0.77–0.81)**	0.90 (0.88–0.93)**	0.95 (0.92–0.98)**	1.11 (1.07–1.15)**
Hispanic, male	0.58 (0.57–0.59)**	0.68 (0.66–0.69)**	0.60 (0.58–0.61)**	0.69 (0.67–0.70)**
Hispanic, female	0.70 (0.69–0.71)**	0.80 (0.78–0.81)**	0.86 (0.84–0.88)**	0.98 (0.95–1.00)*
Ages 8–12				
White, male	Reference	Reference	Reference	Reference
White, female	1.22 (1.16–1.29)**	1.24 (1.18–1.30)**	1.92 (1.80–2.04)**	1.94 (1.82–2.06)**
AAPI, male	0.57 (0.51–0.63)**	0.57 (0.51–0.64)**	0.56 (0.48–0.65)**	0.56 (0.48–0.65)**
AAPI, female	0.97 (0.88–1.06)	0.97 (0.88–1.07)	1.46 (1.31–1.64)**	1.47 (1.31–1.64)**
AI/AN, male	1.05 (0.77–1.44)	1.09 (0.80–1.49)	1.13 (0.74–1.72)	1.20 (0.79–1.82)
AI/AN, female	1.17 (0.87–1.57)	1.24 (0.93–1.67)	1.68 (1.16–2.44)*	1.80 (1.24–2.61)*
Black, male	0.79 (0.73–0.86)**	0.86 (0.80–0.93)**	0.91 (0.82–1.00)	0.99 (0.89–1.1)
Black, female	0.89 (0.82–0.96)*	0.97 (0.90–1.05)	1.41 (1.29–1.55)**	1.54 (1.41–1.69)**
Hispanic, male	0.57 (0.55–0.60)**	0.63 (0.60–0.67)**	0.60 (0.56–0.64)**	0.66 (0.61–0.70)**
Hispanic, female	0.75 (0.72–0.79)**	0.83 (0.79–0.87)**	1.13 (1.06–1.20)**	1.24 (1.16–1.32)**
Ages 13–17				
White, male	Reference	-	Reference	-
White, female	1.62 (1.58–1.67)**	1.65 (1.60–1.69)**	2.21 (2.15–2.29)**	2.25 (2.18–2.32)**
AAPI, male	0.73 (0.69–0.78)**	0.74 (0.70–0.79)**	0.74 (0.69–0.80)**	0.74 (0.69–0.80)**
AAPI, female	1.40 (1.33–1.46)**	1.43 (1.36–1.50)**	2.02 (1.91–2.13)**	2.05 (1.94–2.17)**
AI/AN, male	0.87 (0.71–1.07)	0.94 (0.77–1.16)	1.32 (1.06–1.65)*	1.43 (1.15–1.79)*
AI/AN, female	1.62 (1.40–1.87)**	1.76 (1.53–2.04)**	1.77 (1.50–2.10)**	1.91 (1.61–2.27)**
Black, male	0.64 (0.61–0.68)**	0.73 (0.69–0.77)**	0.64 (0.60–0.68)**	0.74 (0.69–0.78)**
Black, female	1.08 (1.03–1.12)**	1.25 (1.2–01.30)**	1.34 (1.28–1.40)**	1.56 (1.49–1.64)**
Hispanic, male	0.58 (0.57–0.60)**	0.68 (0.66–0.70)**	0.56 (0.54–0.58)**	0.66 (0.64–0.69)**
Hispanic, female	0.92 (0.89–0.94)**	1.07 (1.05–1.11)**	1.19 (1.16–1.23)**	1.41 (1.36–1.45)**
Ages 18–21				
White, male	Reference	-	Reference	-
White, female	0.84 (0.81–0.86)**	0.85 (0.83–0.88)**	0.89 (0.86–0.92)**	0.90 (0.87–0.93)**
AAPI, male	0.76 (0.71–0.80)**	0.76 (0.71–0.81)**	0.73 (0.68–0.79)**	0.73 (0.68–0.79)**
AAPI, female	0.89 (0.85–0.94)**	0.90 (0.86–0.95)**	0.95 (0.89–1.01)	0.96 (0.91–1.03)
AI/AN, male	0.94 (0.77–1.15)	0.98 (0.80–1.20)	0.81 (0.62–1.05)	0.87 (0.67–1.13)
AI/AN, female	0.64 (0.52–0.77)**	0.69 (0.57–0.84)**	0.92 (0.74–1.14)	1.00 (0.80–1.24)
Black, male	0.68 (0.64–0.71)**	0.75 (0.72–0.79)**	0.70 (0.66–0.74)**	0.77 (0.72–0.81)**
Black, female	0.54 (0.51–0.56)**	0.61 (0.58–0.64)**	0.61 (0.58–0.64)**	0.68 (0.65–0.72)**
Hispanic, male	0.63 (0.61–0.65)**	0.71 (0.69–0.73)**	0.65 (0.63–0.68)**	0.72 (0.70–0.75)**
Hispanic, female	0.46 (0.45–0.48)**	0.53 (0.51–0.54)**	0.49 (0.48–0.51)**	0.55 (0.53–0.57)**

* Indicates significance at the *P* < .05 level.

** Indicates significance at the *P* < .001 level.

† Adjusted for insurance type and median household income quartiles

*OR*, odds ratio; *aOR*, adjusted odds ratio; *AAPI*, Asian American Pacific Islander; *AI/AN*, American Indian and Alaska Native.
