# Supplementary material for: Intersectional Analysis of Suicide-related Emergency Department Visits in Youth in California, 2018–2021
Source: West J Emerg Med. 2025 Nov 26;26(6):1611–21. doi: 10.5811/westjem.47097 (PMC12698142; doi:10.5811/westjem.47097)

**Supplemental figure 1a - 1c: Quarterly Trends in Suicide-Related ED Visits among AAPI and White males and females by age group, 2018-2021**

1a. 8- to 12-year-olds


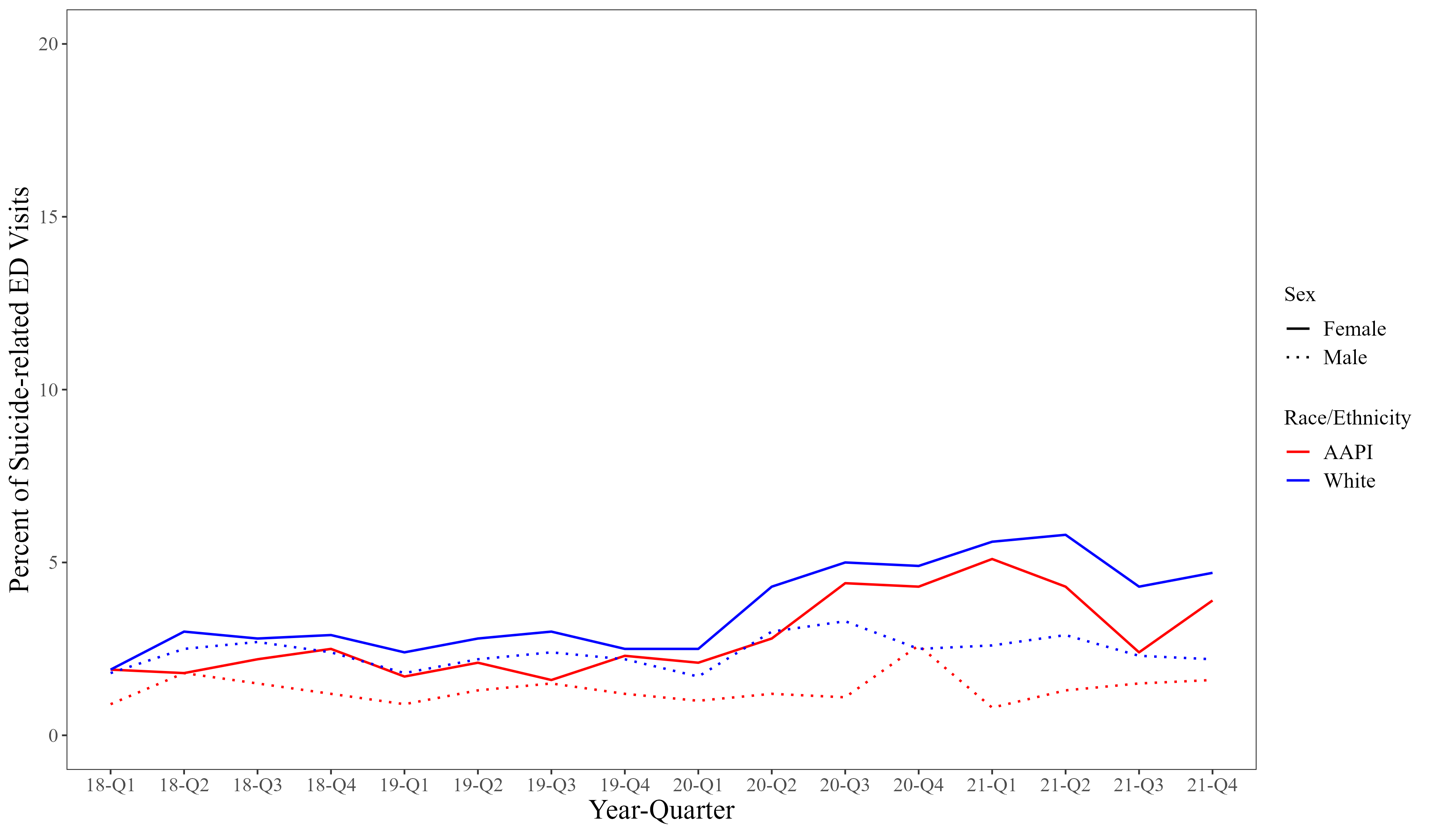


1b. 13- to 17-year-olds


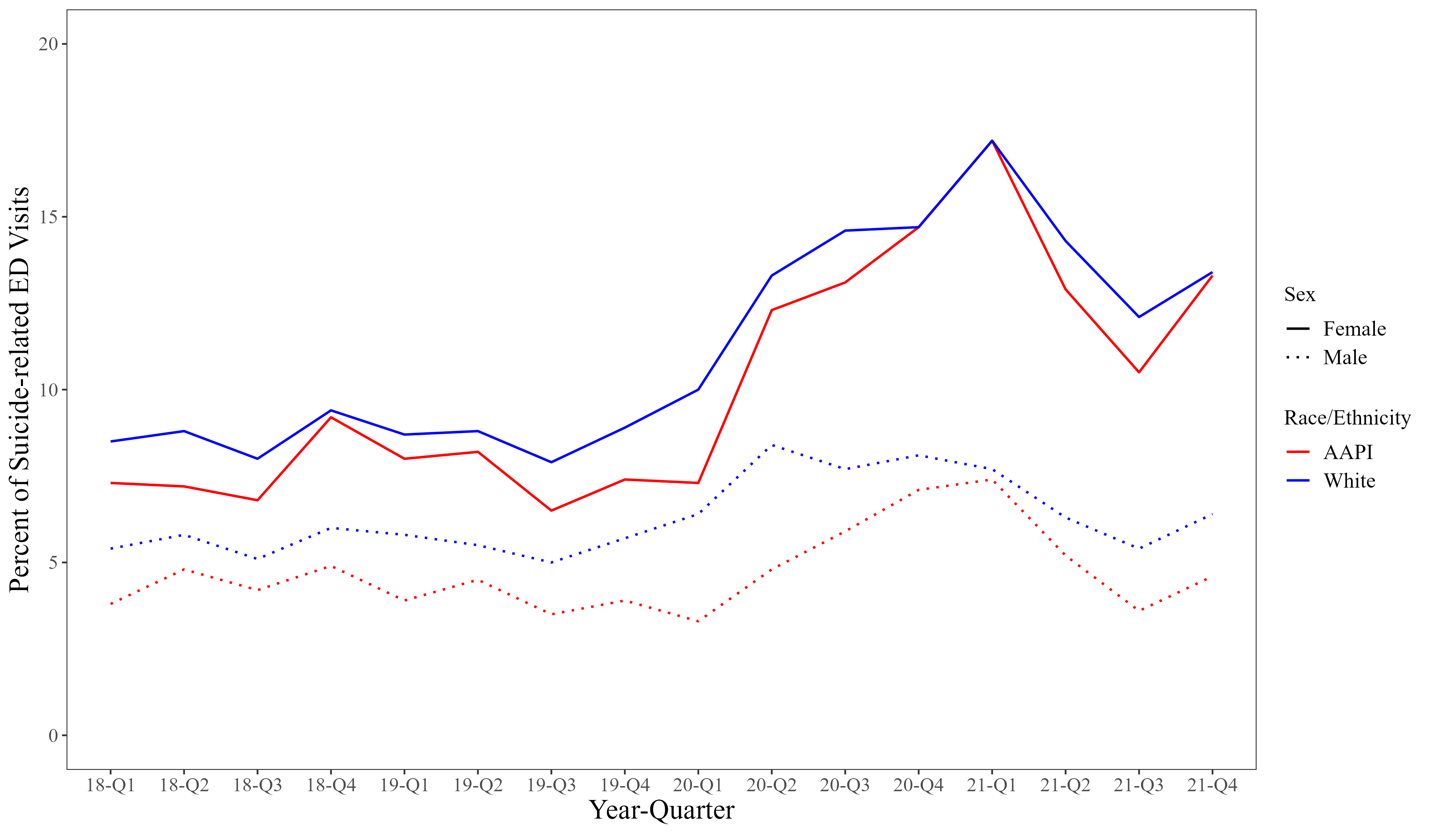


1c. 18- to 21-year-olds


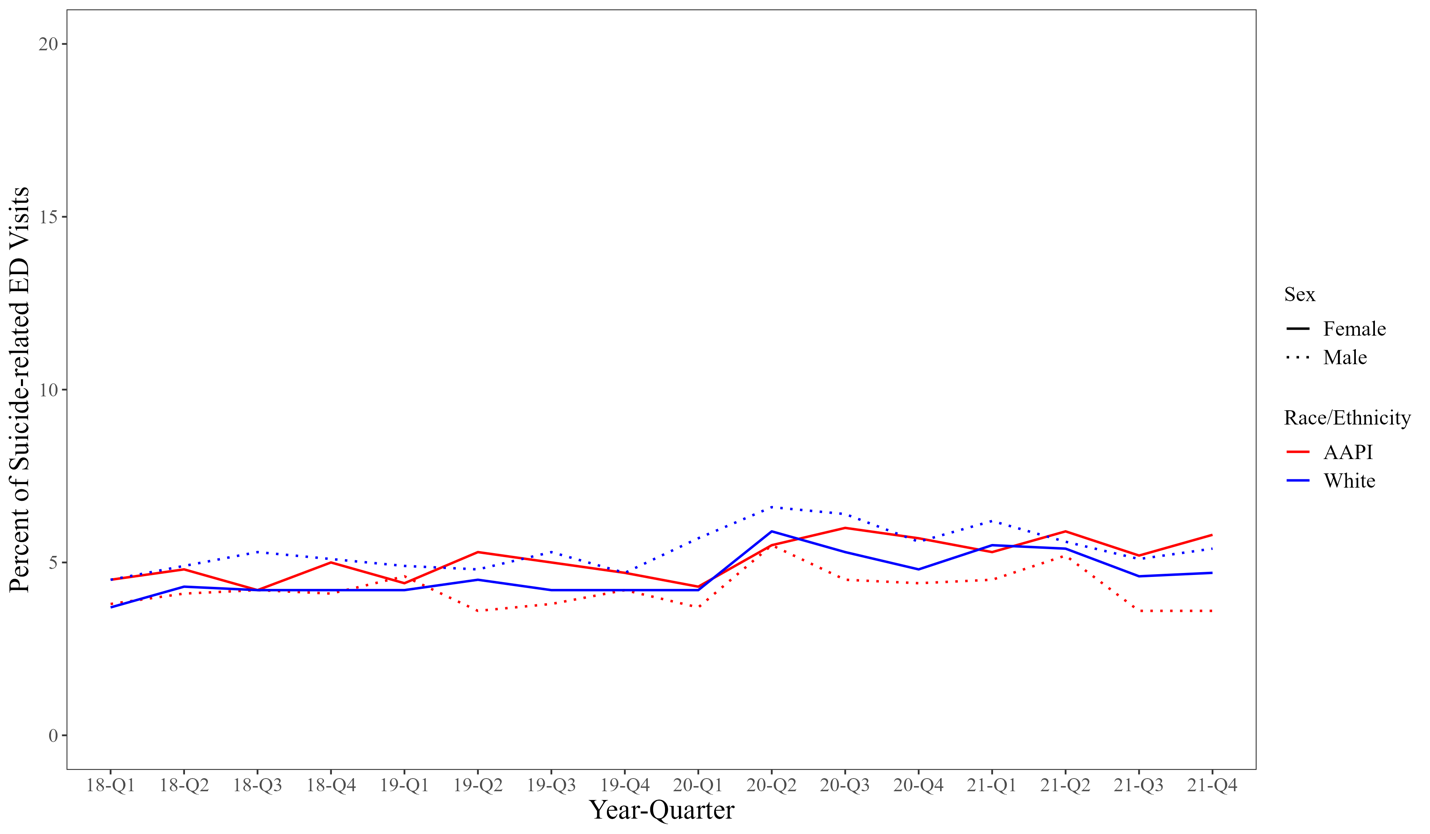

Supplement: Supplementary file 1 [file wjem-26-1611-s001.docx]
