# Supplementary material for: Intersectional Analysis of Suicide-related Emergency Department Visits in Youth in California, 2018–2021
Source: West J Emerg Med. 2025 Nov 26;26(6):1611–21. doi: 10.5811/westjem.47097 (PMC12698142; doi:10.5811/westjem.47097)
Supplement: Supplementary file 2 [file wjem-26-1611-s002.docx]

**Supplementary Table 1. Mortality Rate of Suicide-Related ED Encounter by Age Group, Race/Ethnicity and Sex, and Before/During COVID (2018-2021)**

| **Characteristic** | **Overall**, | **No Deaths**  n (%) | **Deaths**  n (%) | **Mortality rate**^2^ | ***P* value**^3^ |
| --- | --- | --- | --- | --- | --- |
| **N** | 232,762^1^ | 232,631 | 131 |  |  |
| **Sex** |  |  |  |  | **<0.001** |
| Male | 91,133 | 91,046 (39.1) | 87 (66.4) | 9.55 |  |
| Female | 141,629 | 141,585 (60.9) | 44 (33.6) | 3.11 |  |
| **Age (Categorical)** |  |  |  |  | 0.91 |
| 8-12 years | 31,506 | 31,488 (13.5) | 18 (13.7) | 5.71 |  |
| 13-17 years | 116,297 | 116,234 (50.0) | 63 (48.1) | 5.42 |  |
| 18-21 years | 84,959 | 84,909 (36.5) | 50 (38.2) | 5.89 |  |
| **Race/Ethnicity and Sex** |  |  |  |  | **<0.001** |
| White, male | 35,595 | 35,564 (15.3) | 31 (23.7) | 8.71 |  |
| White, female | 56,198 | 56,179 (24.1) | 19 (14.5) | 3.38 |  |
| AAPI, male | 4,957 | 4,950 (2.1) | Suppressed^4^ | 14.12 |  |
| AAPI, female | 8,748 | 8,745 (3.8) | Suppressed^4^ | 3.43 |  |
| Black, male | 8,326 | 8,319 (3.6) | Suppressed^4^ | 8.41 |  |
| Black, female | 13,399 | 13,394 (5.8) | Suppressed^4^ | 3.73 |  |
| Hispanic, male | 41,844 | 41,803 (18.0) | 41 (31.3) | 9.80 |  |
| Hispanic, female | 62,635 | 62,618 (26.9) | 17 (13.0) | 2.71 |  |
| AI/AI, male | 411 | 410 (0.2) | Suppressed^4^ | 24.33 |  |
| AI/AN, female | 649 | 649 (0.3) | Suppressed^4^ | 0.00 |  |
| *Abbreviations*: CI, Confidence Interval; AAPI, Asian American Pacific Islander; AI/AN, American Indian and Alaska Native.  ^1^Excludes 686 missing data on death outcome.  ^2^ Rate per 10,000 people  ^3^Pearson's Chi-squared test  ^4^Counts were suppressed if n<=10 | | | | | |
